# Clinical Outcomes of Patients Managed in a Temporary COVID-19 Step-Down Unit

**DOI:** 10.7759/cureus.99837

**Published:** 2025-12-22

**Authors:** Aaron D Gluth, Jeniffer Carpinello, Jessica Nave, Mary Ann Kirkconnell Hall, David Krakow

**Affiliations:** 1 Division of Hospital Medicine, Emory University School of Medicine, Atlanta, USA; 2 Hospital Medicine, Emory Healthcare, Atlanta, USA

**Keywords:** covid-19, hospital medicine, hospital units, length of stay, transitional care

## Abstract

Background

During the coronavirus disease 2019 (COVID-19) pandemic, unprecedented surges of patients strained healthcare resources, especially intensive care units (ICUs), which were quickly saturated by patients with respiratory failure. To inform future responses, we conducted a retrospective analysis of patient outcome data from a temporary COVID-19 transitional care/step-down unit (TCU) we implemented during January-February 2021.

Methods

Our TCU was embedded on a medical-surgical floor at our academic hospital institution to offload ICU patients with stable or improving respiratory failure, who still required heated humidified high-flow nasal cannula (HHHFNC) and/or noninvasive positive pressure ventilation (NIPPV). Our Hospital Medicine and Critical Care service lines devised specific clinical criteria for patient selection to the unit. We recruited personnel with experience in HHHFNC and NIPPV and ensured that a physician or Advanced Practice Provider was virtually always physically present on the unit. We performed descriptive statistical analysis of patient outcomes (disposition, length of stay, and readmission) and demographic characteristics (age, sex, and baseline comorbidities).

Results

Twenty-six patients were treated in the COVID-19 TCU. At baseline, patients had a mean of 2.8 comorbidities per person (range: 0-7, median 3). Eight patients (31%) were female and 18 (69%) were male. The mean age of the patients was 70.9 years (range: 32-94 years, median 70.5). Five (19.2%) were downgraded to the general medical ward, 17 (65.4%) were discharged to home or another medical facility in good condition, and four (15.4%) were provided comfort care (one died awaiting transport to hospice). The mean TCU length of stay was 7.6 days, and the mean hospital length of stay was 16 days. Only one patient required readmission within one month. No patients experienced unexpected cardiopulmonary arrest or required transfer back to the ICU.

Conclusions

Our TCU, embedded within a medical-surgical floor, effectively and safely liberated ICU beds during a pandemic. Using agreed-upon clinical criteria for appropriate transfer to the TCU and appropriate staffing, we conserved critical care resources and improved patient flow without major adverse events or ICU readmissions.

## Introduction

In late 2019, COVID-19, the disease caused by the novel Severe Acute Respiratory Syndrome Coronavirus 2 (SARS-CoV-2) emerged in Wuhan, China. It was quickly learned that COVID-19 is largely a respiratory illness, with some clinical similarities to SARS-CoV-1 (the virus that causes the Severe Acute Respiratory Syndrome), albeit with higher transmissibility [1]. Wide clinical variability was observed; many patients were asymptomatic or displayed mild flu-like symptoms (81%), while a smaller yet significant portion of infected patients experienced serious illness (14%) or critical illness (5%) [2]. On January 20th, 2020, the first confirmed U.S. case of COVID-19 was documented in Washington state. On January 31, 2020, the COVID-19 outbreak was declared a public health emergency. By the spring of 2020, cases were reported in all 50 states, and several thousand Americans had tested positive for COVID-19 [3,4].

Complex epidemiologic factors led to several subsequent “waves” of patients, which overwhelmed hospitals and stretched acute care resources thin across the US. The sheer number of new patients presenting to the hospital was problematic, but the issue was compounded by the fact that admitted patients with respiratory failure were often slow to wean from high levels of respiratory support. In our hospital system, as in many others, intensive care units (ICUs) became dangerously saturated as a result [5-7]. Previous research had demonstrated that transitioning patients to a step-down unit following ICU discharge was not associated with poorer outcomes than transfer directly to the hospital ward [8]. We hypothesized that a temporary stepdown unit embedded on a medical-surgical floor would allow us to safely decompress our ICUs by offloading relatively stable patients who were still requiring high levels of oxygen therapy.

Though the COVID-19 pandemic is over, the threat of other respiratory pathogens that could rapidly overwhelm ICUs-influenza (avian or other), measles-related acute respiratory distress syndrome, and emergent zoonotic illnesses-remains [9]. Preparedness is still a high priority for health institutions and systems [9]. In this paper, we describe the implementation and outcomes of a temporary COVID-19 transitional care/step-down unit (TCU) implemented during a surge in 2021.

## Materials and methods

Amidst a major COVID-19 surge in January and February of 2021, we implemented a temporary COVID-19 TCU embedded on a medical-surgical floor at our institution (a large academic medical center) to offload ICU patients with stable or improving respiratory failure, who still required heated humidified high-flow nasal cannula and/or noninvasive positive pressure ventilation. It should be noted that our institution does not operate a step-down/intermediate care unit during non-pandemic circumstances.

The TCU was staffed primarily by Hospital Medicine physicians, with close consultative support from our Critical Care Medicine/Pulmonology group. Nocturnal coverage was provided by Hospital Medicine nocturnists and Advanced Practice Providers. Nursing care was provided by medical-surgical nurses who had received in-service training in the use of heated, humidified high-flow nasal cannula. The maximum census of the TCU was eight patients. The nurse-to-patient ratio was maintained at either one-to-two or one-to-three, depending on staffing capabilities. 

**Figure 1:**
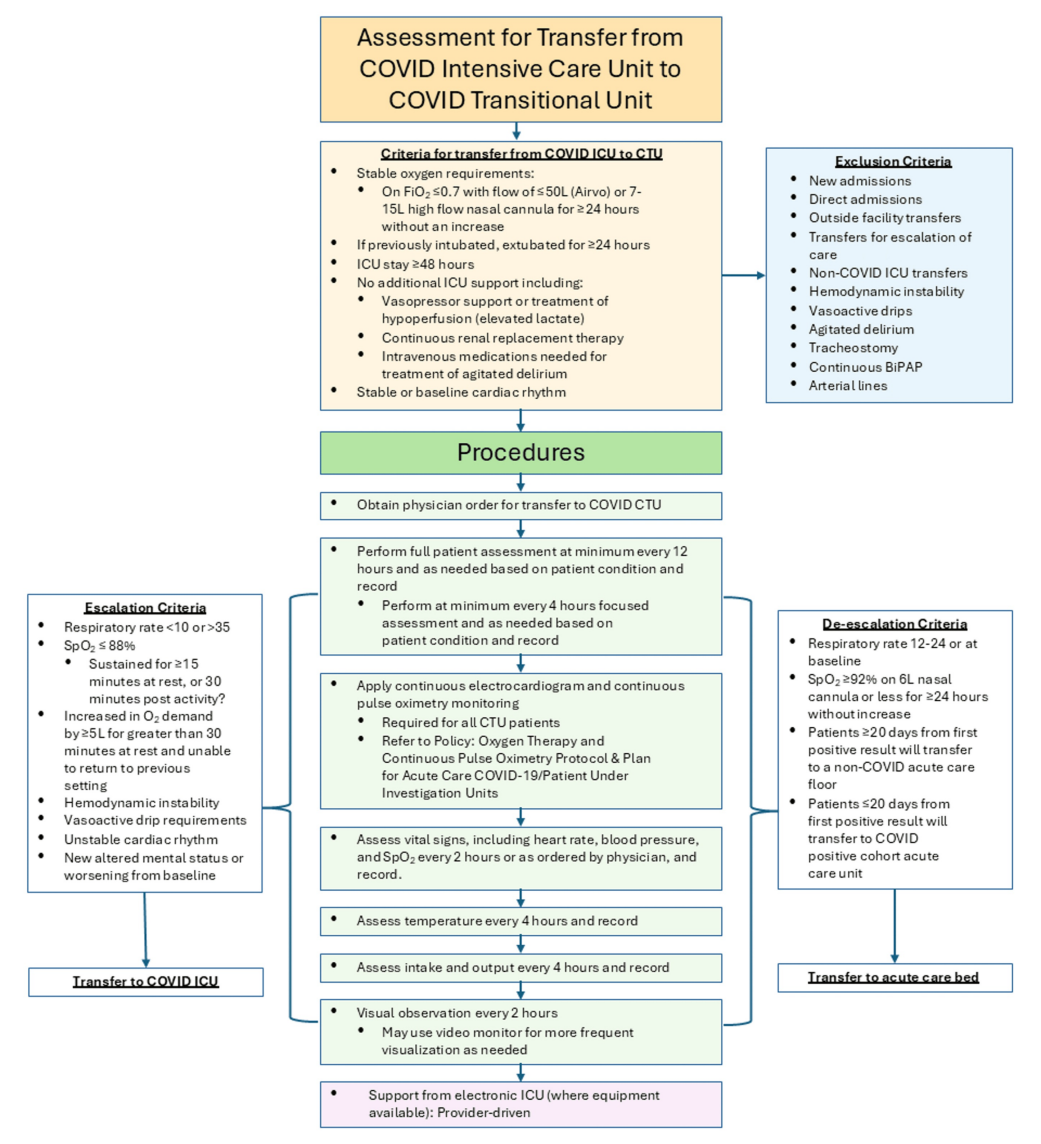
COVID-19 care transition unit criteria and guidelines BiPAP = bilevel positive airway pressure; CTU = COVID transitional/step-down unit; FiO2 = fraction of inspired oxygen; ICU = intensive care unit; Sp02 = peripheral oxygen saturation, COVID-19 = Coronavirus disease 2019.

Hospital Medicine and Critical Care Medicine devised specific clinical criteria for patient selection to the unit (Figure 1). Development of the criteria was primarily led by Critical Care physicians, based both on the limited evidence available in the literature at the time and on observation in a rapidly changing clinical environment. TCU transfer candidates were patients with ongoing respiratory failure, stable on low to moderate amounts of heated humidified high-flow nasal cannula and/or intermittent noninvasive positive pressure ventilation. To ensure stability, patients were accepted only after at least 48 hours in the ICU. If previously ventilated, patients were accepted only after at least 24 hours post-extubation. Exclusion criteria include shock, unstable arrhythmias, agitation, and other acute organ system failure.

ICU patients were selected and reviewed by Critical Care Medicine staff, Hospitalists, and nursing leadership prior to transfer. The TCU operated strictly in a “step-down” fashion; patients were transferred from the TCU only after demonstrating stability in the ICU, and were not accepted from the Emergency Department or from the medical wards. At the time the TCU was instituted, our hospital was operating 153 ICU beds, and 55 of those beds were designated for COVID patients.

Vital signs were assessed every two hours; blood gases were not required but were checked on an as-needed basis when clinically appropriate. Continuous electrocardiogram and continuous pulse oximetry monitoring were used for all patients. Video monitors were used for some patients for more frequent visual observation. Escalation and de-escalation criteria (Figure 1) provided guidance for transferring patients back to the ICU and for downgrading patients to a general medical-surgical ward, respectively. The use of COVID-specific pharmacologic therapies was left to the discretion of the treating physicians, and most patients received dexamethasone, remdesivir, and some form of pharmacologic venous thromboembolism prophylaxis (unfractionated heparin, low molecular weight heparin, direct-acting oral anticoagulants).

We used Microsoft Excel software to conduct descriptive statistical analysis (counts, ranges, and frequencies) of the data. This study was deemed to be quality improvement, not human subjects research, and thus exempt from review by our institution’s Institutional Review Board.

## Results

Twenty-six patients were treated in the COVID-19 TCU during January and February 2021. At baseline, patients had a mean of 2.8 comorbidities per person, with a range: 0-7 and a median of 3 (Table 1). Eight patients (31%) were female and 18 (69%) were male. The mean age of the patients was 70.9 years; the median was similar, at 70.5 years.

**Table 1:** COVID-19 transitional care unit patient characteristics

Baseline characteristics	Values
Mean age, years (range)	70.9 (32-94)
Female sex, n (%)	8 (30.7%)
Race	
White n (%)	15 (57.6%)
Black, n (%)	7 (26.9%)
Asian, n (%)	2 (7.6%)
Hispanic, n (%)	0 (0.0%)
Other/unknown, n (%)	2 (7.6%)
Comorbidities	
Active cancer, n (%)	6 (23.0%)
Obstructive sleep apnea, n (%)	3 (11.5%)
Diabetes Mellitus, n (%)	12 (46.1%)
Chronic obstructive pulmonary disease, n (%)	4 (15.3%)
Interstitial lung disease, n (%)	1 (3.8%)
Asthma, n (%)	1 (3.8%)
Cerebrovascular disease, n (%)	1 (3.8%)
Coronary artery disease, n (%)	6 (23.0%)
Congestive heart failure, n (%)	1 (3.8%)
Dementia, n (%)	4 (15.3%)
Obesity, n (%)	4 (15.3%)
Smoking, n (%)	1 (3.8%)
Hypertension, n (%)	20 (76.9%)
Chronic kidney disease, n (%)	4 (15.3%)
HIV infection, n (%)	0 (0.0%)
Sickle cell disease, n (%)	0 (0.0%)
Transplant, n (%)	2 (7.6%)
Atrial fibrillation, n (%)	2 (7.6%)
Pulmonary hypertension, n (%)	1 (3.8%)

Of those patients treated on the TCU, five patients (19.2%) were downgraded to the general medical ward, 17 patients (65.4%) were discharged to home or another medical facility in good condition, and four patients (15.4%) were provided comfort care; one of these four patients died while awaiting transfer to hospice (Table 2). No patients required return to the ICU or died unexpectedly.

**Table 2:** COVID-19 step-down/transitional care unit patient outcomes

Outcomes		Values
Disposition		
Downgrade to general ward, n (%)	5 (19.2%)	
Return to intensive care unit, n (%)	0 (0.0%)	
Home, n (%)	16 (61.5%)	
Nursing facility, n (%)	1 (3.8%)	
Hospice, n (%)	4 (15.4%)	
Unexpected death, n (%)	0 (0.0%)	
One month readmission, n (%)	1 (3.8%)	
Mean length of stay in step-down/transitional care unit, days (range)	7.6 (2-18)	
Mean total length of stay, days (range)	16 (5-28)	
Complications		
Decompensated heart failure, n (%)	0 (0.0%)	
Acute coronary syndrome, n (%)	0 (0.0%)	
Acute stroke, n (%)	1 (3.8%)	
Shock/bacteremia, n (%)	4 (15.4%)	
Venous thromboembolism, n (%)	3 (11.5%)	
Acute kidney injury, n (%)	7 (26.9%)	
Arrhythmia, n (%)	8 (30.8%)	
Encephalopathy, n (%)	1 (3.8%)	
Intensive care unit maximum respiratory support		
Ventilator, n (%)	4 (15.4%)	
Extracorporeal membrane oxygenation, n (%)	0 (0.0%)	
Noninvasive positive pressure ventilation and/or heated humidified high-flow nasal cannula, n (%)	21 (80.8%)	
Not applicable: no intensive care unit admission, n (%)	1 (3.8%)	
Step-down/transitional care unit respiratory support		
Heated humidified high-flow nasal cannula, n (%)	21 (80.8%)	
Noninvasive positive pressure ventilation, n (%)	7 (26.9%)	
High-flow nasal cannula, n (%)	23 (88.5%)	
Therapeutics		
Remdesivir, n (%)	24 (92.3%)	
Corticosteroids, n (%)	26 (100%)	
Other immunomodulators, n	0 (0.0%)	
Antibiotics, n (%)	19 (73.1%)	

Complications were common, with a mean occurrence of 1.04 complications per patient. Arrhythmia (30.8%) and acute kidney injury (26.9%) were observed most frequently. Most patients were managed both in the ICU and the TCU with an HHHFNC +/- NIPPV, and were then de-escalated to nasal cannula prior to discharge. If patients had been started on nocturnal NIPPV while in the ICU, it was continued after transfer to the TCU. All patients were given steroids, and most received remdesivir (92.3%) and broad-spectrum antibiotics (73.1%). The mean TCU length of stay was 7.6 days, and the mean total hospital length of stay was 16 days. Only one patient required readmission within one month.

## Discussion

Amid a respiratory pandemic, we were able to safely and successfully repurpose part of a med-surg hospital floor as a COVID-19 step-down unit staffed primarily with hospitalist physicians, hospitalist Advanced Practice Providers (i.e., physician assistants and nurse practitioners), and medical-surgical nurses. The implementation of the unit freed up valuable critical care resources for higher-acuity patients and was not associated with any adverse events. Improving patient throughput is likely to improve care delivery and patient outcomes, particularly during a pandemic surge scenario: one recent study noted that hospital quality was inversely related to COVID-19 admission rates [10], and a recent review demonstrated the significant negative impact that ICU strain has on patient outcomes [11]. Agnoletti et al. found that a dynamic model incorporating step-down units was a feasible and effective way to manage patient surges during COVID-19 without compromising patient safety metrics [12].

Our model is based on the careful selection of patients using specific, predetermined criteria. We believe that a key to maintaining safety during the implementation of a TCU is close support from critical care consultants during the development of criteria. Frequent collaboration is also important; in our case, critical care medicine personnel were semi-embedded in the unit and were often physically present and available for consultation whenever necessary.

The TCU and the model’s protocol were tailored for the care of COVID-19 patients with respiratory failure, but we believe a similar care model could be implemented for a variety of other potential epidemic conditions. As noted above, new pathogens continue to emerge and existing ones to evolve, and hospital facilities and systems must be prepared to react quickly when case numbers rise [9,13]. While other pathogens may emerge that are sufficiently different from COVID clinically that our protocol may not be directly applicable in all respects, the resources required to implement a similar TCU quickly are generally available in most hospital settings in the United States [6], and a similar model was effective in a European setting [14]. This is highly salient given the likelihood that many healthcare systems may not only be insufficiently prepared but may also have not yet fully recovered from strains caused by the pandemic [5].

Limitations of our study include the small number of patients, the implementation of the TCU at a single site, and the TCU’s duration of two months. We did not perform economic analyses to determine if the TCU was associated with cost savings, though other studies have noted that step-down units may be associated with lower costs without sacrificing outcomes [15]. In addition, at the time that the TCU criteria (both inclusion and exclusion) were developed, there was a lack of evidence-based guidelines available. Our study’s strengths include our interdisciplinary collaborative care model and its logistical simplicity; it requires no special resources that would preclude or delay implementation during a surge in cases. At the time of publication, we are unaware of any other studies describing the rapid conversion of med-surg beds into a COVID-19 step-down unit within an American hospital.

## Conclusions

Though the COVID-19 pandemic has ended, respiratory pathogens continue to emerge and evolve, and hospitals and systems must be prepared for surges of acutely ill patients. During such surges, ICUs can be quickly overwhelmed. Our model successfully opened up ICU beds without compromising patient outcomes, and its simplicity and reliance on resources that are commonly available in hospitals suggest that it would be effective in other respiratory pandemics and surges. Future research should focus on comparing mortality, length of stay, and cost-effectiveness of critically ill patients stepped down to a temporary step-down unit vs patients managed solely in the ICU.
